# Role of pharmacotherapy in Brugada syndrome

**Published:** 2004-01-01

**Authors:** Takeshi Tsuchiya

**Affiliations:** Division of Cardiology, Hakuaikai Hospital

Brugada syndrome is a unique form of idiopathic ventricular fibrillation (VF), and is characterized by an ECG pattern with right bundle branch block (J wave),  ST-segment elevation and terminal T-wave inversion in leads V1 through V3 [1-10]. It accounts for approximately 20% of all cases of sudden cardiac death in patients with structurally normal hearts and appears to be more frequent in Southeast Asia than in other regions [5].  To date, the only established therapy for preventing sudden cardiac death due to VF in this disease is an implantation of an implantable cardioverter defibrillator (ICD) [1-4].  Although there is no room for discussion regarding the excellent and uniform efficacy of ICDs for terminating VF, the effect is confined to the termination of VF, and ICDs cannot contribute to the prevention of VF.  Therefore, there are some concerns regarding ICD therapy.  The first is electrical storm associated with VF or polymorphic ventricular tachycardia, which is defined as frequent appropriate ICD shock deliveries for ventricular tachyarrhythmias of ≥3 times over 24 hours.  Chalvidan et al [10], reported a patient who suffered from incessant VF episodes (electrical storm) and became near-fatal, but subsequently fully recovered.  The second is ICD related complications including lead dislodgment, inappropriate shock delivery, infection requiring ICD removal and so on) [11-13].  The third is the indication of ICDs for infant cases of Brugada syndrome, in which the size of the ICD is too large for implantation [11-13].  A new therapy other than ICD implantation to overcome these concerns seems to be urgent.

## Cellular mechanism

Brugada syndrome is suggested to be a primary electrical disease, and two dozen mutations of the cardiac sodium channel gene “SCN5A”, which lead to a loss of the channel function, have been found in about 15-20 % of patients with Brugada syndrome, and an autosomal mode of transmission has been demonstrated [4-8].

The ionic and cellular basis for the ST-segment elevation in Brugada syndrome is hypothesized to be due to an outward shift in the balance of the membrane ionic currents at the end of phase 1 and phase 2 of the action potential, in which an outward current is mainly due to the activation of the transient outward current (I_to_), and the inward current is mainly due to the activation of an inward calcium current (I_Ca_) and an inactivating component of an inward sodium current (I_Na_).  The net outward shift of this current balance leads to a loss of the dome or phase 2 of the action potential.  Such changes may affect the right ventricular epicardium more markedly than the endocardium and produce a marked voltage gradient in the membrane potential between the end- and epi-cardial sides of the right ventricular muscle fibers during the phase 2 (a change corresponding to the characteristic ST-segment elevation in V1 through V3 in the ECGs of Brugada Syndrome [4,6-8].

The loss of the action potential dome in the epicardium but not in the endocardium results in the development of a marked transmural dispersion of repolarization and refractoriness, responsible for the development of a vulnerable window during which a premature impulse or extrasystole can induce a reentrant arrhythmia.  Because the loss of the action potential dome in the epicardium is usually heterogeneous, it leads to the development of epicardial dispersion of repolarization and refractoriness.  Conduction of the action potential dome from sites at which it is maintained to sites at which it is lost causes local re-excitation via a phase 2 reentry mechanism, leading to the development of a very closely coupled extrasystole, which captures the vulnerable window across the wall, thus triggering a circus movement reentry in the form of VT or VF [4,6-8].

In patients with Brugada syndrome, an abnormal spike-and-dome configuration of the epicardial monophasic action potentials (MAPs) obtained from the right ventricular outflow tract, may create a prominent J point elevation [14].  Furthermore, a MAP and repolarization of longer duration in the epicardium than in the endocardium, resulting from a delayed dome formation, suggests a reversal current flow during phase 3 of the action potential.  The longer duration of the action potential in the epicardium may have created the typical terminal T-wave inversion observed in Brugada syndrome [14].  Antelevitch et al., [4,7,8] suggested that the arrhythmogenic substrate arises when a further shift in the balance of the current leads to a loss of the action potential dome at some epicardial sites but not others.  Actually, Nagase et al [15]., reported that a delayed potential was observed in the epicardial electrogram of the right ventricular outflow tract but not others, which was considered to be the second upstroke of the epicardial action potential (epicardial echo beat) generated by local and concealed phase 2 reentry [16].

## Pharmacological therapy based on the cellular mechanism

A variety of pathophysiologic conditions and pharmacologic interventions, which either increase the membrane outward currents or reduce the inward currents should produce a loss of the action potential dome in the right ventricular epicardium and facilitate the occurrence of VF.  Phase 2 of the action potential could be abolished by reducing the I_Na_ as predicted by the ST-segment elevation after the application of Class IC agents and restored by increasing the inward current, e.g., by enhancing the ICa or by decreasing the outward I_to_ current, e.g., by the application of quinidine as reported [4,7,8].

 There are many pharmacologic therapies that have been clinically examined. Class IC agents are clearly contraindicated because theses drugs reduce the INa as mentioned above [17]. Class IA agents, including procainamide and disopyramide, are contraindicated since these drugs strongly depress the INa but only mildly affect Ito, and are well known to exacerbate the ST elevation and occurrence of VF [4,7-9].  Class IB agents, which lack an Ito blocking effect and display little use-dependence owing to a rapid dissociation from the sodium channel, are not at all effective in the normalization of the ST-segment elevation [13].  Both amiodarone and beta-blockers are also shown to be ineffective [4,7-9].

Because the presence of a prominent Ito is at the heart of the mechanism underlying Brugada syndrome, any agent that blocks this current in the heart is likely to be protective [4,7,8].  At the time of writing this review, there was no drug developed that could cardio-selectively block the Ito.  An isolated case report showed that DDD-pacing at a relatively high rate is effective in decreasing the I_to_  (and hence suppressing VF), possibly because the repriming time of the I_to_ channels is reported to be fairly long [18].  There is, however, no systematic study to examine the effect of relatively high rate pacing on the prevention of VF.  Quinidine, another class IA agent, has been shown to directly inhibit Ito in addition to a secondary inhibition due to an increase in the heart rate by a vagolytic action [4,7,8].  Experimental studies have shown quinidine to be effective in restoring the epicardial action potential dome, thus normalizing the ST-segment and preventing phase 2 reentry and polymorphic VT in experimental models of Brugada syndrome [7,8].

Belhassen et al. [11], reported very important and provocative observations regarding the effect of quinidine on the prevention of VF in 34 consecutive patents with idiopathic VF, 5 of whom met the diagnostic criterion of Brugada syndrome.  In the study, electrophysiologic study (EP)-guided therapy with class IA agents (mainly quinidine) was performed.  These agents effectively prevented the induction of sustained polymorphic ventricular tachycardia (SPVT) and/or VF in 26 of 27 patients (96%), in all of whom SPVT or VF was induced in the baseline EP studies.  Of the 23 patients treated with these medications, no patients died or had a sustained ventricular arrhythmia during a mean follow-up period of 9.1±5.6 years. They concluded that EP-guided therapy with class IA agents (mainly quinidine) was a reasonable, safe and effective approach for the long-term management of patients with idiopathic VF including Brugada syndrome.  Although there have been no randomized placebo-controlled prospective studies of EP-guided quinidine therapy, I think that quinidine is a promising antiarrhythmic agent.  The experimental antiarrhythmic agent, tedisamil, with its potent Ito, among other outward current, blocking action, has been suggested as a therapeutic candidate [8]. This drug may be more potent than quinidine because it lacks the relatively strong inward current blocking actions of quinidine.

There are, however, some limitations in using quinidine for the prevention of VF in patients with Brugada syndrome.  There are some risks with antiarrhythmic agents with regard to the potential for a loss of efficacy or proarrhythmic effects under a number of conditions including hypokalemia, hypomagnesemia, bradycardia, therapy with other agents that alter repolarization, metabolic inhibitors, and changes in myocardial substrates.  Furthermore, quinidine is known to have other non-cardiac side effects including abdominal cramping, diarrhea, cinchonism, which consists of decreased hearing, tinnitus, and blurred vision, thrombocytopenia, lupus syndrome, and anticholinergic side effects including a dry mouth, urinary retention and so on.    These side effects may affect the compliance with the drug [11]. The anticholinergic action on the heart might contribute to the anti-VF effect of the drug by increasing the heart rate [13].

Another approach for the prevention of VF in patients with Brugada syndrome is to increase the I_Ca_ by promoting an intracellular level of cyclic-AMP.  Intravenous isoproterenol administration is especially known to be effective in suppressing ST elevation in leads V1 through V3 in Brugada syndrome patients, and to restore the action potential dome in models of this syndrome [4,7,8] and patients with Brugada syndrome [9,19], because it markedly increases the ICa secondary to an elevation in the intracellular level of cyclic-AMP.

Cilostazol, a new oral phosphodiesterase type-III inhibitor is primarily used as a strong antiplatelet agent, but has been shown to increase the I_Ca_ and modestly increase the heart rate as well as contractile function. All these effects are secondary to an increased level of the cyclic-AMP caused by an inhibition of phosphodiesterase type-III activity, in guinea-pig ventricular myocytes and papillary muscles [20].  From these findings, it is reasonable to expect that this drug has an antiarrhythmic efficacy for preventing the VF often observed in patients with Brugada syndrome, because it increases the I_Ca_ due to inhibition of the phosphodiesterase activity in the ventricular myocytes and decreases the I_to_ due to an increased heart rate which again is secondary to an increased I_Ca_ in the sinus node. I recently presented a case of a 67-year-old man with Brugada syndrome, in whom daily episodes of ventricular fibrillation occurred early in the morning for 4 days straight and the VF was completely prevented by an oral administration of cilostazol [21]. This effect was confirmed by the on-and-off challenge test, in which the discontinuation of this drug resulted in recurrence of VF, and the resumption of this drug again prevented the VF.  This effect may be related to the suppression of the I_to_ secondary to an increase in the heart rate and/or increase in the I_Ca_ due to an elevation in the intracellular cyclic-AMP concentration via inhibition of phosphodiesterase activity. I performed a long-term follow-up with cilostazol in three patients (the follow-up periods were 12, 26 and 32 months, respectively), and in all an ICD was implanted for the termination of spontaneous VF. In all the patients several VF episodes were observed over a one year period prior to the cilostazol, no VF was recorded by the ICD memory and the patients were free from symptoms under 100-200 mg/day of cilostazol. Further study is needed to examine the effectiveness of the drug.

## Practical way to use anti-VF drugs

In patients who undergo aborted sudden cardiac death or syncope of unknown origin (symptomatic Brugada syndrome), no one argues that the implantation of an ICD is the first-line therapy regardless of the findings of the EP study. For those patients, drug therapy plays not a contradictory but a complimentary role to the ICD by reducing the number of ICD shock deliveries. Prevention of VF contributes to the improvement in the quality of life of the patients by avoiding uncomfortable ICD shock deliveries.

As mentioned above, Belhassen et al. [11], performed EP-guided medical therapy in 34 patients with idiopathic VF, in 5 of whom the criterion of Brugada syndrome were fulfilled, and reported excellent long-term results. Although medical therapy requires markers that can accurately predict the preventive effect of VF over a long-term period, there, however, seems to be no reliable marker.  An EP study is usually used to examine the preventive effects of antiarrhythmic agents on sustained ventricular tachycardia in patients with structural heart disease, whereas the prognostic value of the EP study in predicting life-threatening events in Brugada syndrome is still controversial.  Brugada et al., [3] suggested that among the asymptomatic patients, the inducibility of VT during the EP study might be a prognostic marker of risk.  Studies by Priori et al., [22] and Kanda et al., [23] failed to find an association between the inducibility and recurrence of VT/VF in patients with Brugada syndrome regardless of whether it was symptomatic or asymptomatic.  As Belhassen suggested [13], the difference might be due to the VF induction protocol in the EP study, in which Belhassen et al., used a stimulus current intensity of five times the diastolic threshold along with the use of repetition of double and triple extrastimulation at the shortest coupling intervals that resulted in ventricular capture.  Further appropriate clinical trials are needed to clarify this issue.

In patients with asymptomatic Brugada syndrome who are family members of symptomatic Brugada syndrome patients, the same strategy as that for the symptomatic Brugada syndrome patients should be considered [2-4].  In another asymptomatic-patient group in whom an ECG that discloses the Brugada sign is performed for routine reasons such as a workup prior to surgery or sport license or screening for insurance, risk stratification to find the patients at high risk is needed because in most cases these patients have a benign prognosis [3,22,23]. Antzelevitch et al. [4], recommended that all asymptomatic patients with the Brugada sign should undergo an EP study for risk stratification, and, if inducible, an ICD should be implanted since Brugada et al. [3], reported that an overall 8% life-threatening event rate was found in initially asymptomatic patients.  Belhassen et al., [13] suggested that EP study-guided quinidine therapy might become an alternative to ICD therapy for prophylaxis of arrhythmic events in these patients. Further appropriate clinical trials are needed.
